# Risk preference and choice stochasticity during decisions for other people

**DOI:** 10.3758/s13415-018-0572-x

**Published:** 2018-03-16

**Authors:** Francesco Rigoli, Katrin H. Preller, Raymond J. Dolan

**Affiliations:** 1 City, University of London, London, UK; 2 Wellcome Trust Centre for Neuroimaging, University College London, London, UK; 3 Neuropsychopharmacology and Brain Imaging, Department of Psychiatry, Psychotherapy and Psychosomatics, University Hospital for Psychiatry Zurich, Zurich, Switzerland; 4 Max Planck UCL Centre for Computational Psychiatry and Ageing Research, London, UK

**Keywords:** Social decision-making, Decision-making under risk, Choice stochasticity, Decisions for others, Context effect

## Abstract

In several contexts, such as finance and politics, people make choices that are relevant for others but irrelevant for oneself. Focusing on decision-making under risk, we compared monetary choices made for one’s own interest with choices made on behalf of an anonymous individual. Consistent with the previous literature, other-interest choices were characterized by an increased gambling propensity. We also investigated choice stochasticity, which captures how much decisions vary in similar conditions. An aspect related to choice stochasticity is how much decisions are tuned to the option values, and we found that this was higher during self-interest than during other-interest choices. This effect was observed only in individuals who reported a motivation to distribute rewards unequally, suggesting that it may (at least partially) depend on a motivation to make accurate decisions for others. Our results indicate that, during decision-making under risk, choices for other people are characterized by a decreased tuning to the values of the options, in addition to enhanced risk seeking.

**Electronic supplementary material:**

The online version of this article (10.3758/s13415-018-0572-x) contains supplementary material, which is available to authorized users.

Our decisions usually have their principal consequence for ourselves but often also for other people. There is substantial variation in the relative influence that a choice has on the self and on others (Crockett, Kurth-Nelson, Siegel, Dayan, & Dolan, 2014; Engel, 2011; Engle-Warnick & Slonim, 2004; Everett, Faber, Crockett, & De Dreu, 2015; Fehr & Fischbacher, 2004; Henrich et al., 2010; Nowak, Page, & Sigmund, 2000; Rand & Nowak, 2013; Rand, Greene, & Nowak, 2012; Rand et al., 2014; Ruff & Fehr, 2014; Selten & Stoecker, 1986). Some contexts require decisions on behalf of other people that have no or minimal implications for the self. For instance, in finance the decisions of executive officers have only marginal consequences for themselves as compared to shareholders. Recent research has examined risk taking behavior during choices made for others. Considering conditions involving monetary amounts, a stronger risk aversion has been reported during choices made for the self (choice_S_) relative to choices made for an anonymous individual (choice_O_) (Chakravarty, Harrison, Ernan, & Rutström, 2011; Hsee & Weber, 1997; Mengarelli, Moretti, Faralla, Vindras, & Sirigu, 2014; Pollai & Kirchler, 2012; Pollmann, Potters, & Trautmann, 2014; but see Eriksen & Kvaløy, 2009; Reynolds, Joseph, & Sherwood, 2009). However, aside from risk preferences, other important factors may distinguish these two conditions—for example, choice stochasticity (reflecting the variability of choice with similar decisions). A possibility is that choice stochasticity increases during choice_O_ relative to choice_S_. This could be due to a decreased motivation to make accurate choices during choice_O_ (Engel, 2011), leading to a more frequent sampling of nonpreferred options. Another factor that may account for more stochastic decisions during choice_O_ may be a higher uncertainty about others’ preferences than about one’s own, a factor that may be particularly relevant when the other is an anonymous individual.

In this study, we aimed to elucidate the mechanisms that distinguish choice_S_ and choice_O_ during decision-making under risk. We employed a novel gambling task that allowed us to separate factors reflecting risk preference from factors reflecting choice stochasticity, and to assess their respective role when comparing choice_S_ and choice_O_.

The amount of reward available to other people influences subjective well-being and value-based choice (Boyce, Brown, & Moore, 2010; Clark & Oswald, 1996; Luttmer, 2005; Rutledge, de Berker, Espenhahn, Dayan, & Dolan, 2016). However, the nature of the influence of the reward available to others on choice remains largely unclear. Here, we investigated this by manipulating the reward context (defined as the average reward presented within a block) both for the self and for the other. In previous studies focusing on choice for the self alone, manipulation of the reward context for the self induced participants to consider the same reward amount as more valuable in a low reward context (Kahneman & Tversky, 1979; Kőszegi & Rabin, 2006; Louie, Khaw, & Glimcher, 2013; Martinelli, Rigoli, Dolan, & Shergill, 2018; Rigoli, Chew, Dayan, & Dolan, 2018; Rigoli, Friston, & Dolan, 2016; Rigoli, Friston, Martinelli, et al., 2016; Rigoli, Mathys, Friston, & Dolan, 2017; Rigoli, Rutledge, Chew, et al., 2016; Rigoli, Rutledge, Dayan, & Dolan, 2016; Stewart, 2009; Stewart, Chater, & Brown, 2006). Intuitively, this implies that the fishes caught today will look better if they are more than the fishes caught yesterday. A possibility we analyzed here is that the context for the self and the context for the other play a similar role, predicting that a reward will be considered as more valuable when the context of the self and the context of the other have both low value, as compared to when only one has low value. Intuitively, this would predict that the fishes caught today will look better if they are more than the fishes you caught yesterday, but also more than the fishes another person caught yesterday.

## Method

### Participants

Forty healthy right-handed adults (25 females, 15 males; 20–40 years of age, mean age 24) participated in the study. Such sample size was selected before data collection for performing paired-sample *t* tests to investigate differences between conditions with a medium effect size (Cohen’s *d* = 0.5), and assuming a (two-tailed) significance threshold of .05 and a statistical power of .85 (a procedure that requires 36 participants minimum). All participants had normal or corrected-to-normal vision. None had a history of head injury, a diagnosis of any neurological or psychiatric condition, or was currently on medication affecting the central nervous system. The study was approved by the University College of London Research Ethics Committee. All participants provided written informed consent and were paid for participating. Participants were tested at the Wellcome Trust Centre for Neuroimaging at the University College London.

### Experimental paradigm and procedure

**Fig. 1:**
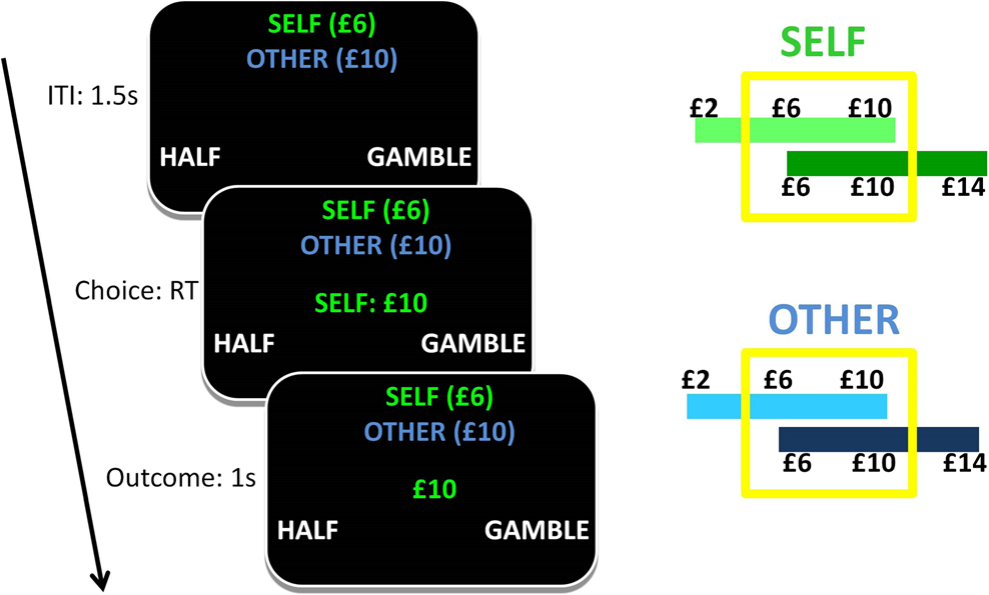
Gambling task. On each trial, a monetary amount (referred as the *trial amount*) was presented, and participants had to choose either half of it for sure (by pressing a left button) or a 50–50 gamble returning either zero reward or the full monetary amount (by pressing a right button). This ensured that the options had equivalent expected values (EVs). In different trials, choice was made either in the interest of the self (choice_S_) or of another participant (choice_O_). The task was organized in short blocks, each comprising ten trials (with five choice_S_ and five choice_O_ trials each). Each block was associated with a context condition that determined the possible EVs associated with the block. The context was manipulated simultaneously in high-value and low-value conditions, relative to both choice_S_ (high_S_ vs. low_S_) and choice_O_ (high_O_ vs. low_O_). This resulted in four conditions for the context: high_S_ & high_O_, high_S_ & low_O_, low_S_ & high_O_, and low_S_ & low_O_. The possible EVs were £1, £3, and £5 for the low-value contexts, and £3, £5, and £7 for the high-value contexts. During an intertrial interval lasting one-and-a-half second, the context condition of both the self and other was signaled by the corresponding average trial amounts (preceded by either the word “self” or “other,” one of which was associated with green text and the other with blue text, with the colors counterbalanced across participants), displayed in brackets at the top of the screen. Possible average trial amounts were £6 and £10 (corresponding to £3 and £5 EV) for the low- and high-value contexts, respectively. Next, the trial amount of the first trial was displayed and choice_S_ or choice_O_ was signaled by the word “self” or “other.” Right after a choice had been made, the outcome appeared for 1 s

Participants performed a computer-based decision-making task lasting approximately 40 min (Fig. 1). On each trial, a monetary amount (referred to as the *trial amount*) that changed trial by trial (600 trials overall) was presented in the center of the screen, and participants had to choose whether to accept half of this amount for sure (by pressing a left button) or to gamble (by pressing a right button). The possible outcomes of the gamble were always either zero reward or the full monetary amount, each with a 50–50 chance. Therefore, on every trial the certain option and the gamble always had the same expected value (EV; corresponding to the sum of all possible outcomes of an option, each multiplied by its probability). We adopted this design because it allowed us to separate factors related to risk preference from factors related to choice stochasticity (see below).

For half of the trials, the choice was made for self-interest (choice_S_). At the end of the experiment, one outcome was randomly selected among those received in choice_S_ trials, and this was paid out to the chooser. For the other half of the trials, the choice was made in the interest of another person (choice_O_), because at the end of the experiment one outcome was randomly selected among those from choice_O_ trials and paid out to the *next* participant involved in the study (and not to the chooser). Specifically, for participant *x* the total payment resulted from averaging an outcome drawn from the choice_S_ trials of that participant and an outcome drawn from the choice_O_ trials of participant *x*–1 (plus a £5 baseline payment). Participants were fully instructed about this payment method. After playing the task, the first participant (unaware of being the first participant in the study) was told that the payment dependent on the other player was £5. Choice_S_ and choice_O_ alternated pseudorandomly and were signaled to participants: On each trial, the trial amount was presented together with either the word “self” or “other” (with the text in either green or blue for self and other, and with the color counterbalanced across participants).

The task was organized in short blocks, each comprising ten trials (five choice_S_ and five choice_O_). Each block was associated with a context condition that determined the possible EVs associated with the block. The context was simultaneously manipulated on the basis of high-value and low-value conditions for both self (high_S_ vs. low_S_) and other (high_O_ vs. low_O_). This resulted in four context conditions: high_S_ & high_O_, high_S_ & low_O_, low_S_ & high_O_, and low_S_ & low_O_. The possible EVs were £1, £3, and £5 for the low-value contexts, and £3, £5, and £7 for the high-value contexts. For example, for the low_S_ & high_O_ condition, the possible EVs were £1, £3, and £5 for choice_S_, and £3, £5, and £7 for choice_O_. The EVs used here were selected because of evidence showing that they elicited an effect of context during choice for the self (Rigoli, Friston, & Dolan, 2016).

The context conditions for both choice_S_ and choice_O_ were signaled by the corresponding average trial amounts (preceded by either the word “self” or “other” in the corresponding text color; see Fig. 1), displayed in brackets at the top of the screen throughout the block. These trial amounts were £6 and £10 (corresponding to £3 and £5 EV) for the low- and high-value contexts, respectively. Before a new block started, the statement “New set” appeared for 2 s, followed by the context condition (average trial amounts), shown for 2 s. Next, the trial amount of the first trial (indicating also the choice_S_ or choice_O_ condition; see above) was displayed, followed immediately after a response by the outcome of the choice, shown for 1 s. The average amounts remained on the screen during an intertrial interval lasting one-and-a-half second. The orders of blocks, context condition, and outcomes were pseudorandomized.

We also assessed situational and personality factors so as to explore a possible link between these factors and any putative difference between choice_S_ and choice_O_. These factors indicated how much one cared about choice_S_ versus choice_O_. After the task, participants indicated (on a 1–5 scale) their motivation to distribute money equally to the self and the other person during the gambling task. Also, participants filled in the Social Dominance Orientation (SDO) questionnaire (Pratto, Sidanius, Stallworth, & Malle, 1994), which captures a preference for hierarchy within the social system and a predisposition toward anti-egalitarianism.

### Model-based analysis

We compared different generative models of choice behavior by estimating separately, for each model considered, the best-fitting parameters for each participant and summing the negative log-likelihoods of the data, given the model and the best-fitting parameters across participants. Parameter estimation was performed using the fminseachbnd function in Matlab.

For model comparison, we compared more complex models with nested models—namely models in which one or more parameters were fixed at zero. To do this, we used the standard approach of the likelihood-ratio test (Casella & Berger, 2002; Daw, 2011), which allows for a comparison of nested models. This analysis is based on the fact that the difference in the negative log-likelihoods times two (2*d*) between a nested and a more complex model follows a chi-square distribution in which the number of degrees of freedom is equal to the number of additional parameters of the more complex model. A chi-square test could then be performed to estimate the probability that the observed *2d* was due to chance, under the null hypothesis that the data were generated by the nested model, allowing for acceptance or rejection of that null hypothesis.

## Results

### Risk preference

**Fig. 2:**
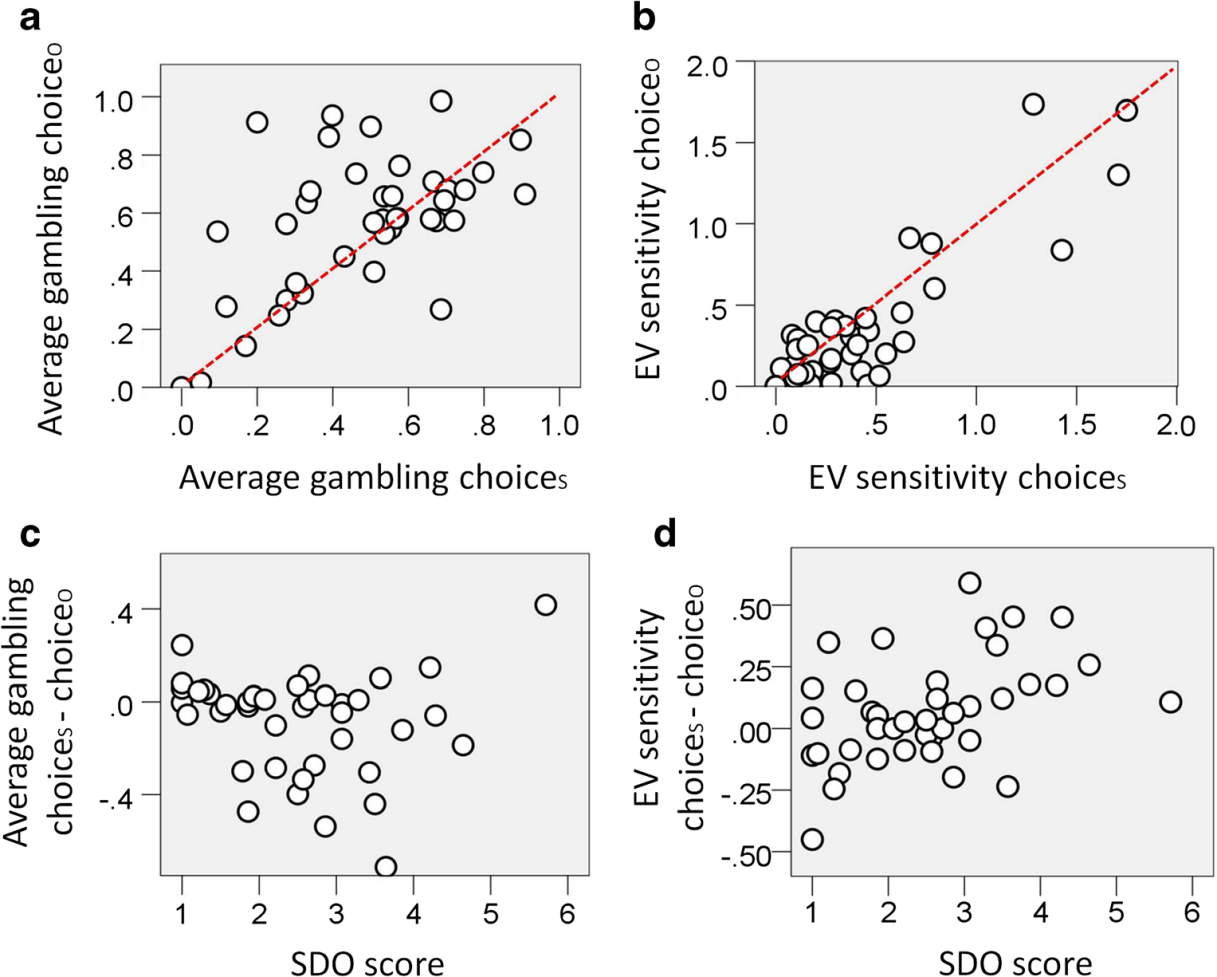
(**a**) Relationship between the proportion of gambling choices during decisions made for the self (choice_S_) and the proportion of gambling choices during decisions made for the other (choice_O_). The red line indicates equal proportions of gambling for choice_S_ and choice_O_. The data show a positive correlation [*ρ*(40) = .591, *p* = .001] and a smaller gambling proportion for choice_S_ than for choice_O_ (*z* = – 2.05, *p* = .040). (**b**) Relationship between EV sensitivity (i.e., the absolute beta weight associated with EV in a logistic regression model of gambling choice) for choice_S_ and EV sensitivity for choice_O_. The red line indicates equal EV sensitivities for choice_S_ and choice_O_. The data show greater gambling sensitivity for choice_S_ than for choice_O_ [*t*(39) = 2.12, *p* = .040]. (**c**) Relationship between score on the Social Dominance Orientation (SDO) questionnaire and the difference in average gambling between choice_S_ and choice_O_ [nonsignificant; *ρ*(40) = – .232, *p* = .149]. (**d**) Relationship between SDO score and the difference in EV sensitivity between choice_S_ and choice_O_ [*ρ*(40) = .458, *p* = .003]

The average gambling proportions were .48 during choice_S_ (*SD* = .23; min = 0, max = .91) and .57 during choice_O_ (*SD* = .24; min = 0, max = 0.99). This resulted in an average gambling proportion that (i) was not different from .5 during choice_S_ [Fig. 2a; *t*(39) = – 0.54, *p* = .59; two-tailed *p* = .05 is used as the significance threshold]; (ii) showed a significance trend toward being greater than .5 during choice_O_ [Fig. 2a; *t*(39) = 1.78, *p* = .082]; and (iii) was smaller for choice_S_ than for choice_O_ (Fig. S1 in suplementary materials; *z* = – 2.05, *p* = .040; for paired-sample comparisons, a *t* test was used if the Shapiro–Wilk test for normality was not significant; otherwise, a Wilcoxon signed rank test was used). The average gambling proportions for choice_S_ and choice_O_ were correlated [Fig. 2a; *ρ*(40) = .591, *p* = .001; Spearman’s correlation was used for our analyses because it is less affected by outliers].

We estimated two logistic regression models of gambling choice (gambling and choice of the certain option were coded as 1 and 0, respectively): one model for choice_S_ and a different model for choice_O_. Each model had the trial EV as a predictor (this was the only predictor in the model; remember that the two options on a trial always had equivalent EVs). Considering each participant individually, the beta weight of the logistic regression associated with EV was significantly different from zero for 27 and 22 participants during choice_S_ and choice_O_, respectively. Across participants, the average beta weights were – 0.025 during choice_S_ (*SD* = 0.61; min = – 1.28, max = 1.75) and – 0.031 during choice_O_ (*SD* = 0.56; min = – 1.74, max = 1.70). This resulted in the beta weight not being different from zero for either choice_S_ [*t*(39) = – 0.256, *p* = .799] or choice_O_ [*t*(39) = – 0.358, *p* = .723], with no difference between the two conditions (Fig. S1 in supplementary materials; *z* = 0.385, *p* = .700). A correlation was evident between the beta weights of the two conditions [Fig. S2 in supplementary materials; *ρ*(40) = .749, *p* < .001].

Standard economic theories postulate that choice results from a nonlinear value function (or an equivalent mean–variance account) mapping an objective reward amount to its underlying subjective value (e.g., Kahneman & Tversky, 1979). In our task (which focuses on gains), such accounts predict that an individual with a concave function will be overall risk-averse and more likely to gamble in small- than in large-EV trials. In addition, a more concave value function would increase risk aversion as well as a preference for gambling in small- as compared to large-EV trials. Conversely, an individual with a convex function will be overall risk-seeking and more likely to gamble with large- than with small-EV trials. A more convex value function would increase risk-seeking and a preference for gambling with large- as compared to small-EV trials. In other words, standard accounts based on a value function predict a correlation across individuals between the overall gambling proportion and the preference to gamble for large versus small EVs. When we tested this prediction in our data, we observed that average gambling and the EV-related beta weight were uncorrelated with each other, both for choice_S_ [*ρ*(40) = .025, *p* = .877] and choice_O_ [*ρ*(40) = – .112, *p* = .491]. This replicated previous findings of ours (Martinelli et al., 2018; Rigoli et al., 2018; Rigoli, Friston, & Dolan, 2016; Rigoli, Friston, Martinelli, et al., 2016; Rigoli, Rutledge, Chew, et al., 2016; Rigoli, Rutledge, Dayan, & Dolan, 2016) and is not explained within the framework of a nonlinear value function, as in standard economic models.

### Choice stochasticity

In addition to examining risk preference, we aimed to explore choice stochasticity (i.e., how much decisions vary in similar conditions) and to assess whether this differed when comparing choice_S_ and choice_O_. This difference can be predicted if, during choice_O_, we hypothesize that agents are less motivated to make accurate decisions or are more uncertain about the other person’s preferences.

We estimated two aspects of choice stochasticity. First, we considered the distance between an individual’s average gambling and 50% gambling (i.e., due to random choices). Across participants, the averages for this measure were 18% during choice_S_ and 20% during choice_O_, with no difference between the two conditions [*t*(39) = – 0.795, *p* = .431]. Second, we computed the absolute beta weight of the logistic regressions associated with EV (see above), which we refer to as *EV sensitivity*. This reports how much choice varies as a function of EV, independent of whether the influence is positive or negative. Note that increased choice stochasticity results in a weaker influence of EV on choice (i.e., a smaller EV sensitivity), because it implies that choice is more variable for similar EVs. Across participants, the average EV sensitivity was larger during choice_S_ (mean = .51) than during choice_O_ (mean = .46) [Fig. 2b and Fig. S1 in supplementary materials; *t*(39) = 2.12, *p* = .040]. These data highlight a difference in choice stochasticity between choice_S_ and choice_O_ that is specific to EV sensitivity.

Comparing the first and second halves of the task, we also analyzed whether time influenced choice behavior or interacted with the effect of self–other condition. However, we found no evidence of any interaction between time and self–other condition, suggesting that the effects of self–other condition did not vary systematically during the task (see the supplementary materials).

### Questionnaires

Our results indicated that, for choice_S_ as opposed to choice_O_, individuals were more attuned to the EV at stake. This effect may be partially dependent on an increased motivation to perform well during choice_S_ as compared to choice_O_. To test this hypothesis, we investigated the relationship between (i) the difference in EV sensitivity for choice_S_ versus choice_O_ and (ii) the difference in how much individuals cared about the self’s versus others’ outcomes. We measured the latter variable with questionnaires about a preference for equality, which by definition captures a difference between caring for the self versus others. Both situational and personality estimates of a preference for equality were collected. The former estimate was assessed through a posttask question in which each participant was asked to indicate (on a 1–5 scale) the motivation to distribute money equally to the self and the other person during the gambling task. Personality factors were assessed by administration of the SDO questionnaire (Pratto et al., 1994; see the Method section), which captures a preference for hierarchy within the social system and a predisposition for anti-egalitarianism. The posttask question score and the SDO score were correlated with each other [*ρ*(39) = – .359, *p* = .025; the score for the posttask question was unavailable for one participant who terminated the task before the end]. The data showed correlations between the difference in EV sensitivity for choice_S_ minus choice_O_ and both the posttask question score [*ρ*(39) = – .377, *p* = .018] and the SDO score [Fig. 2d; *ρ*(40) = .458, *p* = .003].

The correlation analysis left open the question of whether the difference in EV sensitivity for choice_S_ minus choice_O_ was positive for all participants, independent of their posttask question scores (and SDO scores). To address this question, participants were separated into high (score > 3; *n* = 18) and low (score < 4; *n* = 21) posttask question score groups, and a larger EV sensitivity for choice_S_ than for choice_O_ was observed in the low posttask question score group [*t*(20) = 3.50, *p* = .002] but not in the high posttask question score group [*t*(17) = – 0.51, *p* = .960). On the basis of a median split, participants were grouped in high- and low-SDO-score groups, and a larger EV sensitivity for choice_S_ than for choice_O_ was observed for the high-SDO-score group [*t*(19) = 2.96, *p* = .008] but not for the low-SDO-score group [*t*(19) = – 0.192, *p* = .850].

We also examined the relationship between the difference in average gambling for choice_S_ minus choice_O_ and the questionnaire data. We observed no evidence of any relationship of average gambling with the question score [*ρ*(39) = .028, *p* = .866] or with the SDO score [Fig. 2c; *ρ*(40) = – .232, *p* = .149]. In addition, average gambling and EV sensitivity for choice_S_ minus choice_O_ were also uncorrelated with each other [*ρ*(40) = – .031, *p* = .850]. We emphasize that our sample was adequate only for testing large-correlation effect sizes (*ρ* > .5, assuming a power of .8), implying that further research will be needed to test for smaller effect sizes.

### Context effect

In our task, the average trial EV of blocks varied due to a simultaneous manipulation of context for both choice_S_ (high_S_ vs. low_S_) and choice_O_ (high_O_ vs. low_O_). This allowed us to assess whether context exerted an influence on choice behavior for EVs common across different contexts. Thus, in this analysis we investigated the relationship between the EV-related gambling preference (i.e., the beta weight associated with EV of the logistic regression model of gambling) and the difference in gambling for common EVs across low- versus high-value contexts. A positive relationship between these two variables was evident in previous studies (Rigoli et al., 2018; Rigoli, Friston, & Dolan, 2016; Rigoli, Friston, Martinelli, et al., 2016; Rigoli et al., 2017; Rigoli, Rutledge, Chew, et al., 2016; Rigoli, Rutledge, Dayan, & Dolan, 2016), indicating that participants who gambled more with larger EVs also gambled more when the same EVs were relatively large for the context, whereas participants who gambled more with smaller EVs also gambled more when the same EVs were relatively small for the context. This is consistent with a normalization effect exerted by context, because it entails that the very same objective EVs are attributed either higher or lower value, depending on their relative value within the context. However, previous studies had manipulated only a self context and analyzed the choice_S_ condition alone (Rigoli, Friston, & Dolan, 2016; Rigoli, Friston, Martinelli, et al., 2016; Rigoli, Rutledge, Chew, et al., 2016; Rigoli, Rutledge, Dayan, & Dolan, 2016), and the impact of the average contextual reward for a choice made on behalf of another person remained an open question. Here, by manipulating the context for both choice_S_ and choice_O_, we could address this question. Our initial prediction was that the context of the self and the context of the other would exert similar influences and that these influences would involve both choice_S_ and choice_O_. For example, this reasoning implies that, during both choice_S_ and choice_O_, the same EV would be considered more valuable when low_S_ and low_O_ both applied than when either applied alone. As above, we emphasize that our sample was adequate only for testing large correlation effect sizes (*ρ* > .5, assuming a power of .8), implying that further research will be needed to test for smaller effect sizes in the face of null correlation effects found here (see below).

**Fig. 3:**
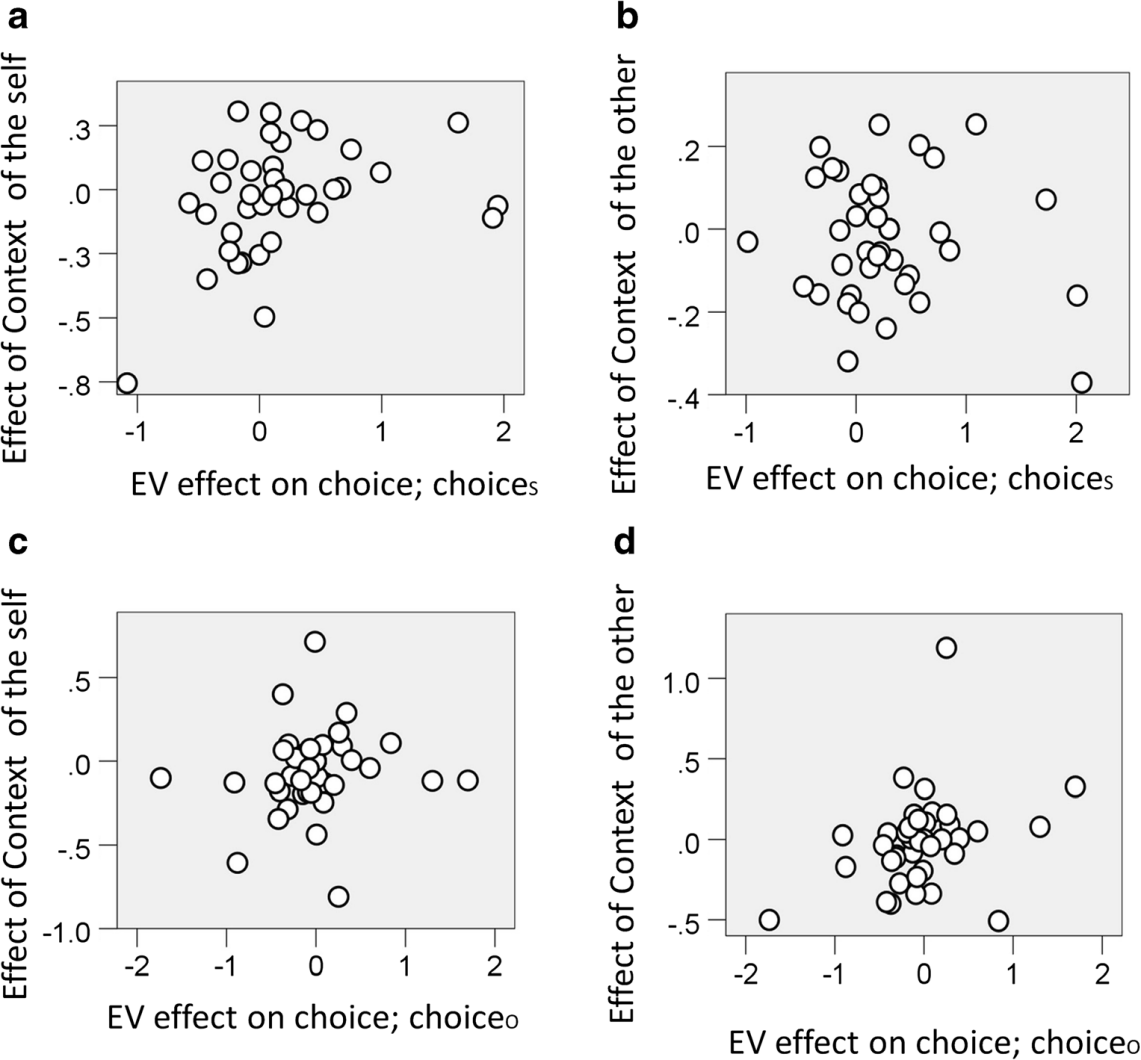
(**a**) Considering choices made for the self (choice_S_), relationship between (i) the effect of EV on gambling preference (i.e., the beta weight associated with the EV of the logistic regression model of gambling for choice_S_) and (ii) the effect of the context of the self, equal to the difference in gambling for common EVs in a low-value context of the self (low_S_) versus a high-value context of the self (high_S_) [*ρ*(40) = .381, *p* = .015]. (**b**) Again considering choice_S_, relationship between (i) the effect of EV on gambling preference and (ii) the effect of the context of the other, equal to the difference in gambling for common EVs in a low-value context of the other (low_O_) versus a high-value context of the other (high_O_) [nonsignificant; *ρ*(40) = – .024, *p* = .884]. (**c**) Considering choices made for the other (choice_O_), relationship between the effect of EV on gambling preference (this time estimated with a logistic regression model of gambling for choice_O_) and the effect of context of the self [nonsignificant; *ρ*(40) = .193, *p* = .232]. (**d**) Again considering choice_O_, relationship between the effect of EV on gambling preference and the effect of context of the other [*ρ*(40) = .381, *p* = .015]

For choice_S_, we observed a correlation between the EV-related gambling preference (i.e., the beta weight associated with EV of the logistic regression model of gambling for choice_S_) and the difference in gambling for common EVs in low_S_ versus high_S_ contexts (independent of the context condition for choice_O_) [Fig. 3a; *ρ*(40) = .318, *p* = .045]. This replicated previous findings (Rigoli, Friston, & Dolan, 2016; Rigoli, Friston, Martinelli, et al., 2016; Rigoli, Rutledge, Chew, et al., 2016; Rigoli, Rutledge, Dayan, & Dolan, 2016) showing an effect consistent with a value normalization exerted by the self context on choice_S_. However, considering choice_S_, no correlation emerged between the EV-related gambling preference and the difference in gambling for common EVs in low_O_ versus high_O_ contexts (independent of the context condition for choice_S_) [Fig. 3b; *ρ*(40) = – .024, *p* = .884]. There was no correlation, either, between the EV-related gambling preference and gambling for the interaction between self and other context (i.e., [low_self_ – high_self_] – [low_other_ – high_other_]) [*ρ*(40) = .156, *p* = .335]. This indicates that choice_S_ was not affected by the other person’s context. This suggests that during choice_S_, the same EV was not perceived as more valuable during low_O_ than during high_O_, which is inconsistent with our initial prediction.

For choice_O_, we observed a correlation between the EV-related gambling preference (this time estimated with a logistic regression model of gambling for choice_O_) and the difference in gambling for common EVs in low_O_ versus high_O_ contexts (independent of the context condition for choice_S_) [Fig. 3d; *ρ*(40) = .381, *p* = .015]. However, again considering choice_O_, there was no correlation between the EV-related gambling preference and the difference in gambling for common EVs in low_S_ versus high_S_ contexts (independent of the context condition for choice_O_) [Fig. 3c; *ρ*(40) = .193, *p* = .232]. No correlation emerged, either, between the EV-related gambling preference and gambling for the interaction between self and other context (i.e., [low_self_ – high_self_] – [low_other_ – high_other_]) [*ρ*(40) = – .114, *p* = .484]. This suggests that during choice_O_, the same EV was not perceived as more valuable during low_S_ than during high_S_, which is also inconsistent with our initial prediction.

Overall, these observations indicate that the context of the self affects choice_S_ but not choice_O_, whereas the context of the other person has a similar influence, but on choice_O_ and not choice_S_.

### Model-based analysis

We deployed the same computational model as in our previous study (Rigoli, Friston, & Dolan, 2016; Rigoli, Friston, Martinelli, et al., 2016; Rigoli, Rutledge, Chew, et al., 2016; Rigoli, Rutledge, Dayan, & Dolan, 2016) to characterize the mechanisms underlying choice behavior (see the Method section). As compared to the versions used before, here we extended the model to account for the influence of the contexts of both self and other. The goal of the model-based analysis was to provide insight into the computations underlying the effects found above, especially in relation to EV sensitivity and the influence of context. Specifically, the model provides a clear formalization of EV sensitivity, cast in terms of how much choice is influenced by the options’ variance, and provides a clear definition of the influence of context, cast in terms of subtractive normalization (see below). The model was inspired by a standard mean–variance return account [in which the value of an option *x* is *V*(*x*) = mean(*x*) + *α* variance(*x*)], with the inclusion of a further bias effect linked to a disposition to gamble. Taking *A* as the sure monetary outcome (received by choosing half of the trial amount), the value of the sure option is *V*_SURE_ = *A*, and the value of the gamble is *V*_GAMB_ = *A* + *α* A^2^ + *μ*. A value function parameter *α* determines whether the reward variance was attractive (*α* > 0) or not (*α* < 0), and a gambling bias parameter *μ* determines a baseline propensity to gamble, capturing whether gambling was attractive (*μ* > 0) or not (*μ* < 0). The probability of choosing the gamble is given by a sigmoidal choice rule *σ*(*V*_GAMB_ − *V*_CERT_) = 1/[1 + exp(−*V*_GAMB_ + *V*_SURE_)]. The role of each parameter is explained in Fig. S3 in supplementary materials, illustrating choice behavior for simulated agents with different parameter sets. Note the model implies that *V*_GAMB_ − *V*_CERT_ = *α* A^2^ + *μ*. This is analogous to a simple logistic regression having the value function parameter *α* as its slope and the gambling bias parameter *μ* as its intercept, where the value of the sure option *A* corresponds to the trial EV (Rigoli, Friston, & Dolan, 2016; Rigoli, Friston, Martinelli, et al., 2016; Rigoli, Rutledge, Chew, et al., 2016; Rigoli, Rutledge, Dayan, & Dolan, 2016). In other words, the computational model is similar to the simple logistic regression adopted above, and the value function parameter *α* is similar to the EV-related parameter in the logistic regression. An implication is that the absolute value of *α* is expected to capture EV sensitivity, and hence will differ when comparing choice_O_ and choice_S_ and show a relationship with the questionnaire measures.

First we assessed whether this model (including both the gambling bias parameter *μ* and the value function parameter *α*) was better than simpler models in which either *μ* or *α* was fixed at zero. A likelihood ratio test showed that our model was favored over a random model [Table 1; Model 4 vs. Model 1: *χ*^2^(80) = 4,778, *p* < .001], a model with *α* = 0 [Table 1; Model 4 vs. Model 2: *χ*^2^(40) = 4,026, *p* < .001] and a model with *μ* = 0 [Table 1; Model 4 vs. Model 3: *χ*^2^(40) = 3,592, *p* < .001]. Note that a model with *μ* = 0 (and with *α* alone as a free parameter) is the one predicted by standard economic theories of choice, proposing that a value function alone is sufficient to explain choice behavior. Contrary to these theories, this analysis shows that both a gambling bias *μ* and a value function parameter *α* drove choice behavior in our task. This is also consistent with the observation of a lack of correlation between the average gambling and the EV-dependent gambling (i.e., the beta weight associated with the EV of the logistic regression model) reported above. In addition, this result suggests that participants overall felt that their choices were consequential, since their behavior was dependent on the reward at stake (as is evident from the selection of a model with *α*). This is also consistent with the results of the logistic regression analysis of choices reported above, showing that 27 participants (for choice_S_) and 22 participants (for choice_O_) had a beta weight associated with an EV significantly different from zero.

**Table 1:** Computational models of choice behavior

Model	Free Parameter	*N* Free Parameters	Neg LL	Pseudo-*R*^2^
1	Random	0	15,249	0
2	*μ*	1	14,873	.106
3	*α*	1	14,656	.119
4	*μ α*	2	12,860	.227
5	*μ*_*S*_ *μ*_*O*_*α*	3	12,211	.266
6	*μ α*_*S*_ *α*_*O*_	3	12,261	.263
7	*μ*_*S*_ *μ*_*O*_ *α*_*S*_ *α*_*O*_	4	12,094	.273
8	*μ*_*S*_ *μ*_*O*_*α*_*S*_ *α*_*O*_ *τ* (effect of *φ*_*S*_ in all trials)	5	12,044	.276
9	*μ*_*S*_ *μ*_*O*_ *α*_*S*_ *α*_*O*_ *τ* (effect of *φ*_*O*_ in all trials)	5	12,061	.275
10	*μ*_*S*_ *μ*_*O*_ *α*_*S*_ *α*_*O*_ *τ* (effect of *φ*_*S*_ in choice_S_; effect of *φ*_*O*_ in choice_O_)	5	12,011^**^	.278
11	*μ*_*S*_ *μ*_*O*_ *α*_*S*_ *α*_*O*_ *τ* (effect of both *φ*_*S*_ and *φ*_*O*_ in all trials)	5	12,046	.276
12	*μ*_*S*_ *μ*_*O*_ *α*_*S*_ *α*_*O*_ *τ*_*S*_ *τ*_*O*_ (effect of *φ*_*S*_ in choice_S_; effect of *φ*_*O*_ in choice_O_)	6	11,995	.279

The second column indicates the free parameters, the third column indicates the number of free parameters per subject. The fourth column indicates the negative log likelihood (Neg LL) of the choice data, given the model and the estimated parameters. The model selected by model comparison (Model 10) is marked with asterisks. The fifth column reports pseudo-*R*^2^, a quantity that indicates the absolute variability explained by the model

Second, we investigated whether different value function parameters *α* and gambling bias parameters *μ* were used for choice_S_ and choice_O_. We considered a model implementing *α*_S_ and *μ*_S_ for choice_S_ and *α*_O_ and *μ*_O_ for choice_O_ and, on the basis of a likelihood ratio test, this model was favored to a model in which *α*_S_ = *α*_O_ [Table 1; Model 7 vs. Model 5: *χ*^2^(40) = 234, *p* < .001]; to a model in which *μ*_S_ = *μ*_O_ [Table 1; Model 7 vs. Model 6: *χ*^2^(40) = 334, *p* < .001]; and to a model in which both *α*_S_ = *α*_O_ and *μ*_S_ = *μ*_O_ [Table 1; Model 7 vs. Model 4: *χ*^2^(80) = 1,532, *p* < .001].

Third, we probed the computational mechanisms underlying the effect of context, using *φ*_*S*_ = 1 and *φ*_*S*_ = 0 to indicate high_S_ and low_S_, respectively, and *φ*_*O*_ = 1 and *φ*_*O*_ = 0 to indicate high_O_ and low_O_, respectively. We compared the following models that implemented different influences of context. One model (Table 1: Model 8) prescribed that, for both choice_O_ and choice_S_ trials, only the context of the self *φ*_*S*_ counted, implying *V*_SURE_ = *A* − *τφ*_*S*_ and *V*_GAMB_ = *A* − *τφ*_*S*_ + *α* (*A* − *τφ*_*S*_)^2^ + *μ*. Another model (Table 1: Model 9) prescribed that, for both choice_O_ and choice_S_ trials, only the context of the other *χ*_*O*_ counted, implying *V*_SURE_ = *A* − *τφ*_*O*_ and *V*_GAMB_ = *A* − *τφ*_*O*_ + *α* (*A* − *τφ*_*O*_)^2^ + *μ*. Another model (Table 1: Model 10) prescribed that the context of the self *φ*_*S*_ counted for choice_S_ and the context of the other *φ*_*O*_ counted for choice_O_. Another model (Table 1: Model 11) prescribed that both the context of the self *φ*_*S*_ and the context of the other *φ*_*O*_ counted for all trials, so that *V*_SURE_ = *A* − *τ*[(*φ*_*S*_ + *φ*_*O*_)/2] and *V*_GAMB_ = *A* − *τ*[(*φ*_*S*_ + *φ*_*O*_)/2] + *α*(*A* − *τ*[(*φ*_*S*_ + *φ*_*O*_)/2])^2^ + *μ*. Given that these models all had equal numbers of parameters (i.e., *α*_S_, *α*_O_, *μ*_S_, *μ*_O_, and *τ*), the favored model was simply the one with the smallest negative log-likelihood. This turned out to be the model in which the context of the self *φ*_*S*_ counted for choice_S_ and the context of the other *φ*_*O*_ counted for choice_O_ (Table 1: Model 10). In addition, a likelihood ratio test showed that this model was favored to a simpler (nested) model in which *τ* = 0 [Table 1; Model 10 vs. Model 7: *χ*^2^(40) = 166, *p* < .001] and to a model in which two different context parameters were implemented (*τ*_S_ for choice_S_ and *τ*_O_ for choice_O_) but that was equivalent otherwise [Table 1; Model 12 vs. Model 10: *χ*^2^(40) = 32, *p* = .812].

The model favored by the model comparison (Table 1: Model 10) included *α*_S_ (median value: .064), *α*_O_ (median value: .877), *μ*_S_ (median value: – .024), *μ*_O_ (median value: – .014), and *τ* (median value: .47) as free parameters and prescribed that the context of the self *φ*_*S*_ counted for choice_S_ and the context of the other *φ*_*O*_ counted for choice_O_. As we explained above, the value function parameter *α* was expected to be analogous to the EV-related weight of the logistic regression model of gambling (see above). Replicating previous findings (Rigoli, Friston, & Dolan, 2016; Rigoli, Friston, Martinelli, et al., 2016; Rigoli, Rutledge, Chew, et al., 2016; Rigoli, Rutledge, Dayan, & Dolan, 2016), the data confirmed that these two measures were highly correlated [*ρ*(40) = .92, *p* < .001 for choice_S_; *ρ*(40) = .93, *p* < .001 for choice_O_]. In addition, the difference between the absolute values of *α*_S_ and *α*_O_ (analogous to the difference in EV sensitivity) was larger than zero (*z* = 2.06, *p* = .040) and was correlated with the posttask question [*ρ*(39) = – .362, *p* = .018] and with SDO scores [*ρ*(40) = .432, *p* = .006].

To validate our model comparison further, we also performed control analyses on data simulated with the model (reported in the supplementary material). Collectively, these analyses demonstrated that the model favored by model comparison replicated the main behavioral findings, supporting the idea that it captures key mechanisms involved in our task.

## Discussion

Investigating decision-making for the interest of somebody else is important to understanding complex social situations. Decreased risk aversion has been observed during monetary choices made for an anonymous individual (Chakravarty et al., 2011; Hsee & Weber, 1997; Mengarelli et al., 2014; Pollai & Kirchler, 2012; Pollmann et al., 2014; but see Eriksen & Kvaløy, 2009; Reynolds et al., 2009). We extended this literature examining the specific contributions of risk preference and choice stochasticity. Comparing choice_S_ versus choice_o_, we found lower average gambling and increased EV sensitivity (i.e., choices being more dependent on the EV at stake). The latter finding highlights a difference in one aspect related to choice stochasticity, in that a decreased EV sensitivity implies a higher choice variability for similar EVs.

The difference in EV sensitivity could arise from the fact that the motivation to make appropriate choices may be stronger during choice_S_ than during choice_o_ (Engel, 2011). In line with this, we observed a correlation between the difference in EV sensitivity and situational (motivation to distribute money equally as reported in a post-task question) and personality (SDO score) variables indicating a preference for an equal reward distribution. In other words, choice behavior of individuals with low (state and trait) motivation to distribute money equally was more tuned to the EVs at stake during choice_S_ than during choice_o_, reflected in an increased EV sensitivity in the former condition. Also, these data hint that a decreased motivation during choice_O_ than during choice_S_ is not ubiquitous but arises out of situational dispositions and personality traits, which in turn are likely to be connected to cultural factors. Considering constructs related to SDO, such as social value orientation (capturing a tendency to distribute resources equally; Van Lange, 1999) and self-reported altruism (Rushton, Chrisjohn, & Fekken, 1981), an interesting question is whether these constructs play any role in the effect on EV sensitivity found here when comparing choice_S_ and choice_O_. These constructs may explain additional variance of the effect, or even mediate the relationship between SDO and the effect.

A second factor that may contribute to the difference in EV sensitivity depends on a lack of information about the other person. This implies that, during choice_O_ as compared to choice_S_, participants were likely to be more uncertain about the preferences of the other person than about their own preferences, and hence they were more uncertain about whether or not to gamble with different EVs. Our study did not aim to assess the role of uncertainty about others, and further research is needed to elucidate the role played by this factor during choices made for other individuals.

Although choice_O_ and choice_S_ differed in terms of EV sensitivity, the distance between average gambling and 50%—which is another index of choice stochasticity—was not different across conditions. The finding of a specific effect on EV sensitivity can be potentially explained calling upon the notion of motivation but also of uncertainty. One can argue that tuning choice to the EV at stake on a trial-by-trial basis (expressed in the EV sensitivity) requires higher motivation than does establishing whether or not gambling is a good strategy overall (expressed in the distance from 50% gambling). This can explain why a difference in motivation between choice_O_ and choice_S_ translates to a specific difference in EV sensitivity (being the latter the aspect most affected by motivation). Alternatively, one can argue that the evaluation processes engaged to establish when to gamble as a function of EV (underlying the EV sensitivity) are more complex than the processes engaged to establish whether gambling is overall a good strategy or not (underlying the distance from 50% gambling). This would imply that uncertainty on another individual’s preferences would impact especially on EV sensitivity (assuming one is more uncertain about more complex processes), predicting higher EV sensitivity during choice_S_ than during choice_O_.

Like some previous studies, in our task participants were not given information about the person they were choosing on behalf, an aspect important when evaluating the ecological validity of our results. We note many important ecological scenarios in which this information is scarce, usually because the decision is made on behalf of several other people. For example, in finance and politics, information on individual shareholders and voters, respectively, is minimal (a manager knows almost nothing about the specific utility function or risk preference of each individual shareholder). We argue that our task mimic these scenarios in which the decision-maker makes choice on behalf of another person and has scarce knowledge on her individual preferences. In other circumstances, information about the other person is available. Previous literature has shown that, when choosing on behalf of another person, the decision-maker takes into considerations the other person’s preferences inferred on the basis of the available information (Daruvala, 2007).

Most previous studies adopting monetary payoffs have observed an increased risk aversion during choices for the self than for choices for an anonymous individual (Chakravarty et al., 2011; Hsee & Weber, 1997; Mengarelli et al., 2014; Pollai & Kirchler, 2012; Pollmann et al., 2014; but see Eriksen & Kvaløy, 2009; Reynolds et al., 2009). However, previous literature has not examined separately the contribution of a baseline gambling propensity (corresponding to the average gambling proportion) and a gambling preference dependent on EV (corresponding to the *signed* beta weight related to EV in a logistic regression model of choice). These two measures were orthogonal in our task, enabling us to assess their specific contribution. When comparing choice_S_ versus choice_o_, decisions were characterized by a reduced baseline gambling propensity, but gambling did not increase for larger EVs nor it increased for smaller EVs (i.e., the signed EV-related beta weight did not differ). Though our study is not informative on why a difference in baseline gambling emerges, previous research suggests some possibilities. Recent studies have highlighted a baseline risk propensity factor independent of the EV at stake (Rigoli, Rutledge, Chew, et al., 2016; Rutledge, Skandali, Dayan, & Dolan, 2015). Such a baseline risk propensity may reflect an individual bias for the subjective probability of the best outcome of a gamble (Rigoli, Rutledge, Chew, et al., 2016). This would imply an increased subjective probability attributed to the best outcome of the gamble during choices made for other people, resulting in an inflated optimism bias in this condition (Sharot, Guitart-Masip, Korn, Chowdhury, & Dolan, 2012).

The distribution of reward in a particular context influences value attribution and choice, entailing that the very same reward can be perceived as more valuable in a low-reward context (Kahneman & Tversky, 1979; Kőszegi & Rabin, 2006; Louie et al., 2013; Martinelli et al., 2018; Rigoli et al., 2018; Rigoli, Friston, & Dolan, 2016; Rigoli, Friston, Martinelli, et al., 2016; Rigoli, Mathys, Friston, & Dolan, 2017; Rigoli, Rutledge, Chew, et al., 2016; Rigoli, Rutledge, Dayan, & Dolan, 2016; Stewart, 2009; Stewart et al., 2006). In addition to the individual context, living with other people creates social contexts (determined by the reward distribution available to others) that also might influence how an individual evaluates rewards and makes choice. In our task, the context of the self affected choice_S_ but not choice_O_, while the context of the other person affected choice_O_ but not choice_S_. This extends previous findings showing that individuals take the context of another person into account during choice_O_, indicating the reward for others is evaluated relative to the context.

Previous studies have shown that other people’ reward affected subjective well-being and value-based choice (Boyce et al., 2010; Clark & Oswald, 1996; Luttmer, 2005; Rutledge et al., 2016). However, our data did not show any evidence for an influence of the context of the other during choice_S_ (though we emphasize that further research is required to test for smaller effect sizes). This might be explained by the fact that the context of another person influences an individual’s own choices only when the context of the self and other are dependent, as in previous studies (Blake et al., 2015; Blanco, Engelmann, & Normann, 2011; Charness & Rabin, 2002; Engelmann, 2012; Fehr & Schmidt, 1999; Rutledge et al., 2016). Conversely, a lack of influence may characterize conditions under which the two contexts are independent, as in our task. In other words, these data raise the possibility that an impact of the reward available to other people on choice_S_ and well-being should be expected only when the context of the self and of the other are interdependent, for example when differences are perceived as unfair or when the level of reward of others is thought to affect the level of reward for the self.

In sum, we show that individuals are more tuned to the option features during choice_S_ than during choice_o_, and that this effect correlates with trait and state variables capturing a motivation to distribute rewards equally or unequally. We also observed that individuals are more attracted by risk during choice_o_ than during choice_S_. Finally, we found the context of the self affects choice_S_ but not choice_O_, whereas the context of the other person affects choice_O_ but not choice_S_. This indicates that in our task participants segregate reward representations for self and for other, and raises the possibility that context of the other may affect choice_S_ only if the context of the self and the context of the other are interdependent. The findings highlight processes that impact choices made for other people, and this may have implications for how decisions are made in social contexts such as in finance.

## Electronic supplementary material

ESM 1 (DOCX 489 kb)

ESM 2 (DOCX 15 kb)
