# Supplementary material for: Risk preference and choice stochasticity during decisions for other people
Source: Cogn Affect Behav Neurosci. 2018 Mar 16;18(2):331–41. doi: 10.3758/s13415-018-0572-x (PMC5889416; doi:10.3758/s13415-018-0572-x)
Supplement: Supplementary file 1 — (DOCX 489 kb) [file 13415_2018_572_MOESM1_ESM.docx]

**SUPPLEMENTARY MATERIAL**

**Analyses on the effect of time**

An interesting question is whether the differences in choice behaviour comparing choice_S_ and choice_O_ are stable or interact with time given the high number of trials. To investigate this, we compared choice behaviour in the first versus second half of the task. We first analysed the relationship between choice behaviour during the first and second half of the task, and found a correlation for average gambling (choice_S_: ρ(40) = 0.882, p < 0.001; ρ(40) = 0.791, p < 0.001), for the effect of EV on choice (i.e., the beta weight of the logistic regression associated with EV; choice_S_: ρ(40) = 0.882, p < 0.001; choice_O_: ρ(40) = 0.696, p < 0.001), for EV sensitivity (the absolute value of the beta weight; choice_S_: ρ(40) = 0.611, p < 0.001; choice_O_: ρ(40) = 0.521, p = 0.001), and for choice consistency (see main text on how this index is defined; choice_S_: ρ(40) = 0.685, p < 0.001; choice_O_: ρ(40) = 0.582, p = 0.001). Next, we run 2x2 repeated-measure ANOVA analyses having self versus other and time (first versus second half of the task) as factors. For average gambling, we observed a main effect of self versus other (F(1,39) = 6.35, p = 0.016), no main effect of time (F(1,39) = 0.24, p = 0.624) and no interaction (F(1,39) = 1.49, p = 0.230). For the beta weight relative to the effect of EV on choice, we observed no main effect of self versus other (F(1,39) = 0.23, p = 0.634), no main effect of time (F(1,39) = 0.23, p = 0.634) and no interaction (F(1,39) = 1.84, p = 0.183). For EV sensitivity, we observed a main effect of self versus other (F(1,39) = 9.13, p = 0.004), no main effect of time (F(1,39) = 0.78, p = 0.382) and no interaction (F(1,39) = 0.72, p = 0.400). For choice consistency, we observed no main effect of self versus other (F(1,39) = 0.21, p = 0.649), a main effect of time (F(1,39) = 16.65, p < 0.001) and no interaction (F(1,39) = 0.41, p = 0.526). The main effect of time was driven by a lower choice consistency in the first compared to the second part of the task (t(39) = -4.08, p < 0.001).

Overall, these analyses indicate that, except for choice consistency, time does not affect in any systematic manner the expression of choice behaviour. In addition, time does not interact with the self and other choice conditions for any variable. This indicates that the effects of self versus other on average gambling (decreasing during choice_S_ compared to choice_O_) and on EV sensitivity (increasing during choice_S_ compared to choice_O_) do not differ in a systematic way over the course of the task.

**Control analyses of the model comparison**

The model favoured by model comparison (tab. 1: Model 10) includes α_S_, α_O_, µ_S_, µ_O_ and τ as free parameters and prescribes that the context of the self $\varphi_{S}$counts for choice_S_ and the context of the other $\varphi_{O}$ counts for choice_O_. We used this model and subject-specific parameters estimates to generate simulated data and then perform behavioural analyses on the simulated data. First, we investigated the relationship between the real and simulated data, and found a correlation for both the average gambling proportion (choice_S_: ρ(40) = 0.98845, p < 0.001; choice_O_: ρ(40) = 0.98849, p < 0.001) and the effect of EV on choice (i.e., the beta weight from the logistic regression model) (choice_S_: ρ(40) = 0.94113, p < 0.001; choice_O_: ρ(40) = 0.94198, p < 0.001).

Second, we compared the analyses of the simulated data with the analyses of the real data. Consistent with real data, the model replicated (i) a decreased average gambling for choice_S_ compared to choice_O_ (t(39) = -2.27, p = 0.028), (ii) an increased EV sensitivity for choice_S_ compared to choice_O_ (t(39) = 2.09, p = 0.042), (iii) for choice_S_, a correlation between the EV-related gambling preference (estimated with a logistic regression model of gambling for choice_S_) and the difference in gambling for common EVs comparing low_S_ versus high_S_ contexts (ρ(40) = 0.395, p = 0.012), (iv) for choice_O_, a correlation between the EV-related gambling preference (estimated with a logistic regression model of gambling for choice_O_) and the difference in gambling for common EVs comparing low_O_ versus high_O_ contexts (ρ(40) = 0.413, p = 0.008), (v) for choice_S_, a lack of correlation between the EV-related gambling preference (estimated with a logistic regression model of gambling for choice_S_) and the difference in gambling for common EVs comparing low_O_ versus high_O_ contexts (ρ(40) = 0.122, p = 0.451), (vi) for choice_O_, a lack of correlation between the EV-related gambling preference (estimated with a logistic regression model of gambling for choice_O_) and the difference in gambling for common EVs comparing low_S_ versus high_S_ contexts (ρ(40) = -0.131, p = 0.420). Point (i) was not replicated when using an equivalent model except that µ_S_ = µ_O_ (t(39) = 1.41, p = 0.168); point (ii) was not replicated when using an equivalent model except that α_S_ = α_O_ (t(39) = 0.11, p = 0.914); point (iii) was not replicated when using an equivalent model except for the prescription that the context of the other $\varphi_{O}$ alone exerted an influence in all trial (tab 1: Model 9) (ρ(40) = -0.121, p = 0.457); point (iv) was not replicated when using an equivalent model except for the prescription that the context of the self $\varphi_{S}$ alone exerted an influence in all trial (tab 1: Model 8) (ρ(40) = 0.021, p = 0.897); point (v) and point (vi) were not replicated when using an equivalent model except for the prescription that the context of the self $\varphi_{S}$ and the context of the other $\varphi_{O}$ both exerted an influence in all trials (tab 1: Model 11) (point (v): ρ(40) = 0.387, p = 0.014; point (vi): ρ(40) = 0.421, p < 0.007). Collectively, these analyses demonstrate that the model favoured by model comparison replicates the main behavioural findings, supporting the idea that it captures key mechanisms involved in our task.


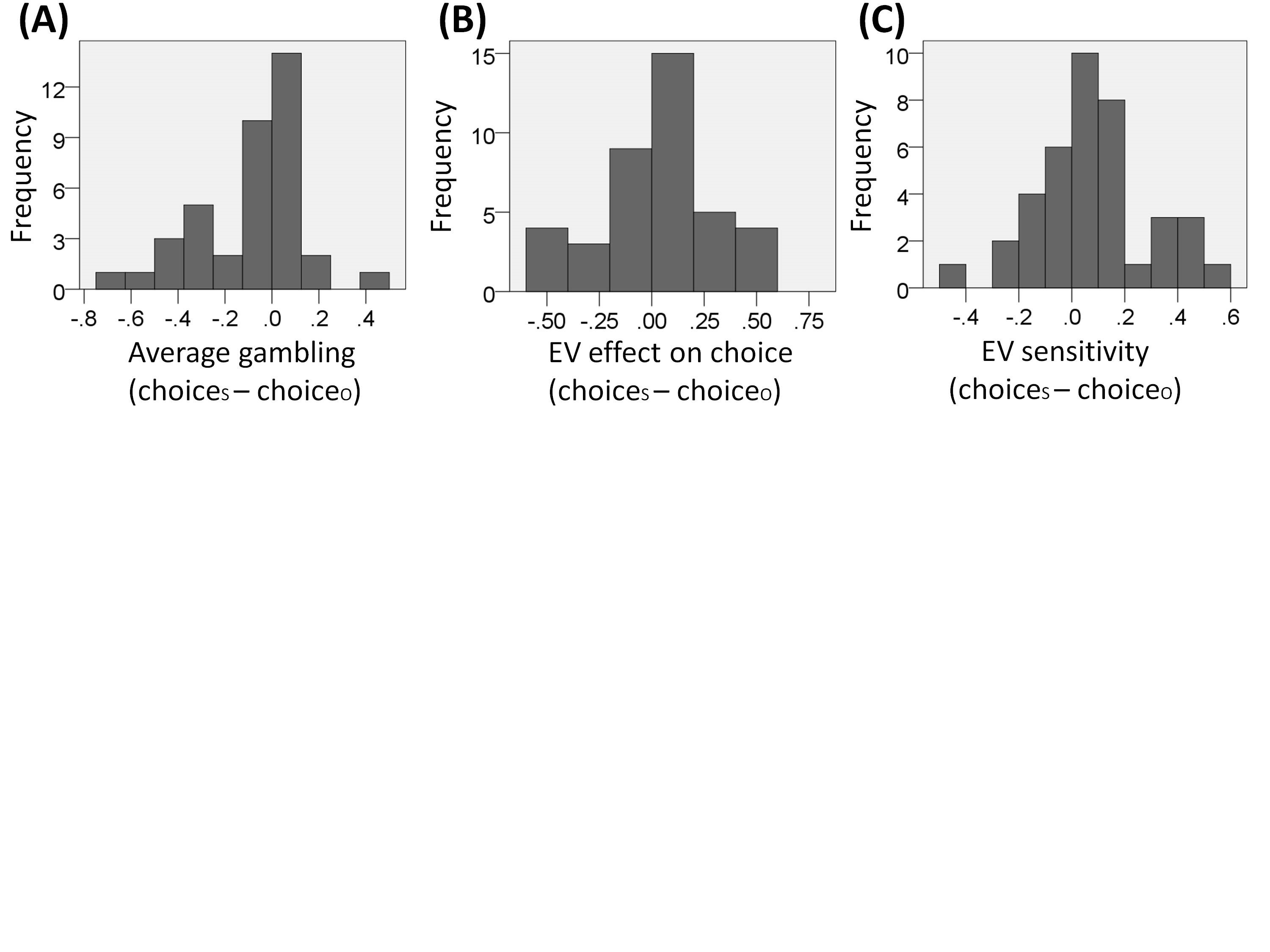


**Fig. S1**. Distribution of the difference between choice_S_ and choice_O_ in the experimental sample (n = 40) for (**A**) average gambling, (**B**) the effect of EV on choice (i.e. the beta weight of the logistic regression associated with EV) and (**C**) EV sensitivity (i.e., the absolute value of the beta weight above).


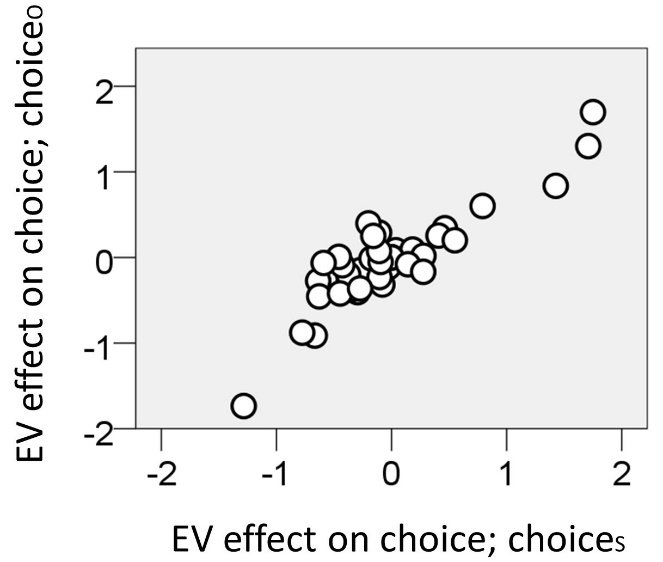


**Fig. S2**. Relationship between the beta weights (reflecting the effect of EV on choice) for choice_S_ and choice_O_ (ρ(40) = 0.749, p < 0.001). The beta weights were computed in two separate logistic regression models of choice, one for choice_S_ and the other for choice_O_.


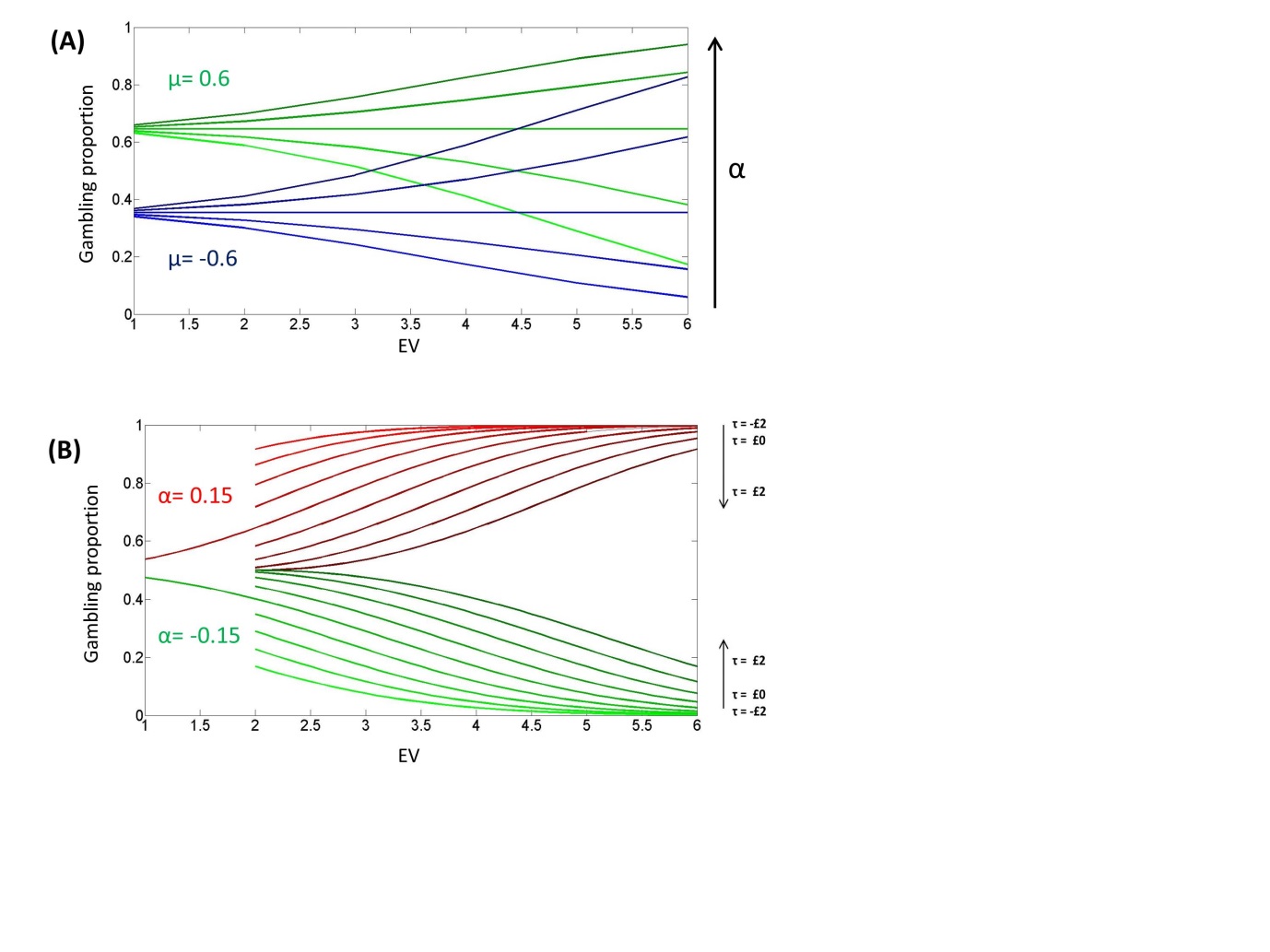


**Figure S3.** Plots of the gambling probability as a function of trial EV (remember that the two options always had equivalent EV) for a set of simulated agents with specific parameters. **A:** Effect of varying the value function parameter α (from $£^{-1}$-0.06 to $£^{-1}$ 0.06 with increases in $£^{-1}$0.03 steps, represented along a bright-to-dark gradient) and the gambling bias parameter μ (green and blue lines implement μ=£0.6 and μ=-£0.6 respectively). It is evident that α determines the tendency to gamble for large or small amounts, while μ is analogous to an intercept parameter reflecting the tendency to gamble for an hypothetical EV of zero. Here the context parameter τ is set to zero **B:** Effect of varying the value function parameter α and the context parameter τ. Red lines represent agents with a positive value function coefficient α (equal to $£^{-1}$0.15) and green lines represent agents with a negative alpha (equal to $£^{-1}$-0.15). Behaviour of agents with τ equal to zero is represented by lines extending from EV=£1 to EV=£6. For the high-value context extending from EV=£2 to EV=£6, agents with different τ are plotted in which τ increases in £0.5 steps from -£2 to £2 along a bright-to-dark gradient.
