# Supplementary material for: Risk preference and choice stochasticity during decisions for other people
Source: Cogn Affect Behav Neurosci. 2018 Mar 16;18(2):331–41. doi: 10.3758/s13415-018-0572-x (PMC5889416; doi:10.3758/s13415-018-0572-x)
Supplement: Supplementary file 2 — (DOCX 15 kb) [file 13415_2018_572_MOESM2_ESM.docx]

**DATA MATRIX**

| **Mean Gamb S** | **Mean GambO** | **EV Effect S** | **EV Effect O** | **EV Sens S** | **EV Sens O** | **Context effect of S on choice S** | **Context effect of S on choice O** | **Context Effect of O on choice S** | **Context Effect of O on choice O** | **Mean Gamb S minus O** | **EV Sens S minus O** | **SDO** |
| --- | --- | --- | --- | --- | --- | --- | --- | --- | --- | --- | --- | --- |
| 0.69 | 0.99 | -0.37 | -0.31 | 0.37 | 0.31 | 0.31 | -0.32 | 0.1 | -0.1 | -0.3 | 0.07 | 1.79 |
| 0.12 | 0.28 | 0.04 | 0.08 | 0.04 | 0.08 | -0.1 | -0.07 | -0.25 | -0.34 | -0.16 | -0.05 | 3.07 |
| 0.28 | 0.3 | -0.09 | -0.13 | 0.09 | 0.13 | 0.09 | 0.25 | 0.04 | -0.08 | -0.02 | -0.04 | 2.57 |
| 0.43 | 0.45 | 1.75 | 1.7 | 1.75 | 1.7 | -0.1 | -0.37 | -0.12 | 0.33 | -0.02 | 0.05 | 1.86 |
| 0 | 0 | 0 | 0 | 0 | 0 | 0 | 0 | 0 | 0 | 0 | 0 | 1.86 |
| 0.67 | 0.57 | -0.08 | -0.32 | 0.08 | 0.32 | 0.04 | -0.06 | -0.29 | -0.11 | 0.1 | -0.24 | 3.57 |
| 0.67 | 0.71 | -0.03 | -0.11 | 0.03 | 0.11 | 0.19 | -0.24 | -0.09 | 0.15 | -0.04 | -0.09 | 1.5 |
| 0.1 | 0.54 | -0.27 | -0.15 | 0.27 | 0.15 | 0.07 | 0.08 | -0.2 | 0.01 | -0.44 | 0.12 | 3.5 |
| 0.7 | 0.68 | -0.64 | -0.27 | 0.64 | 0.27 | -0.1 | -0.16 | -0.09 | -0.27 | 0.02 | 0.36 | 1.93 |
| 0.32 | 0.32 | -1.28 | -1.74 | 1.28 | 1.74 | -0.8 | -0.03 | -0.1 | -0.5 | 0 | -0.45 | 1 |
| 0.57 | 0.58 | 0.18 | 0.09 | 0.18 | 0.09 | -0 | -0.11 | -0.13 | 0.17 | -0.01 | 0.09 | 3.07 |
| 0.8 | 0.74 | -0.3 | -0.4 | 0.3 | 0.4 | -0.1 | 0.03 | -0.18 | 0.04 | 0.06 | -0.11 | 1 |
| 0.5 | 0.9 | -0.35 | -0.37 | 0.35 | 0.37 | -0.3 | -0.16 | 0.4 | -0.4 | -0.4 | -0.03 | 2.5 |
| 0.72 | 0.57 | -0.63 | -0.45 | 0.63 | 0.45 | -0.4 | 0.2 | -0.13 | -0.04 | 0.15 | 0.17 | 4.21 |
| 0.05 | 0.02 | -0.11 | 0.29 | 0.11 | 0.29 | 0.3 | 0.1 | 0.09 | 0.09 | 0.03 | -0.18 | 1.36 |
| 0.46 | 0.74 | 0 | 0 | 0 | 0 | 0 | 0 | 0 | 0 | -0.27 | 0 | 2.71 |
| 0.4 | 0.94 | -0.2 | 0.4 | 0.2 | 0.4 | -0.3 | -0.05 | 0.01 | 0.01 | -0.54 | -0.2 | 2.86 |
| 0.69 | 0.64 | -0.67 | -0.91 | 0.67 | 0.91 | 0.11 | 0.12 | -0.13 | 0.03 | 0.05 | -0.25 | 1.29 |
| 0.51 | 0.4 | 0.79 | 0.6 | 0.79 | 0.6 | 0.07 | 0.25 | -0.04 | 0.05 | 0.11 | 0.19 | 2.64 |
| 0.55 | 0.55 | 1.71 | 1.3 | 1.71 | 1.3 | -0.1 | -0.16 | -0.12 | 0.08 | 0.01 | 0.41 | 3.29 |
| 0.58 | 0.76 | 0.28 | 0.02 | 0.28 | 0.02 | -0.1 | -0.18 | -0.1 | 0.11 | -0.19 | 0.26 | 4.64 |
| 0.54 | 0.64 | 0.46 | 0.34 | 0.46 | 0.34 | 0.01 | -0.01 | 0.29 | -0.09 | 0.01 | 0.12 | 2.64 |
| 0.54 | 0.66 | -0.38 | -0.2 | 0.38 | 0.2 | -0.3 | -0.18 | 0.02 | 0.04 | -0.12 | 0.18 | 3.86 |
| 0.33 | 0.63 | -0.43 | -0.09 | 0.43 | 0.09 | -0.2 | -0.09 | -0.18 | -0.34 | -0.3 | 0.34 | 3.43 |
| 0.53 | 0.58 | 1.43 | 0.84 | 1.43 | 0.84 | 0.26 | 0.07 | 0.11 | -0.51 | -0.05 | 0.59 | 3.07 |
| 0.57 | 0.58 | 0.41 | 0.26 | 0.41 | 0.26 | 0 | 0.17 | 0.17 | 0.15 | -0.01 | 0.15 | 1.57 |
| 0.91 | 0.66 | -0.17 | -0.01 | 0.17 | 0.01 | -0.1 | -0.09 | 0.71 | -0.2 | 0.24 | 0.16 | 1 |
| 0.39 | 0.86 | -0.1 | -0.23 | 0.1 | 0.23 | -0.2 | -0.06 | 0.02 | 0.38 | -0.47 | -0.12 | 1.86 |
| 0.26 | 0.25 | 0 | 0 | 0 | 0 | 0 | 0 | 0 | 0 | 0.01 | 0 | 2.07 |
| 0.66 | 0.58 | -0.1 | -0.05 | 0.1 | 0.05 | -0 | 0.08 | -0.19 | -0.01 | 0.08 | 0.04 | 1 |
| 0.51 | 0.57 | -0.46 | 0.01 | 0.46 | 0.01 | 0.12 | 0.14 | -0.44 | 0.31 | -0.06 | 0.45 | 4.29 |
| 0.9 | 0.85 | 0.55 | 0.2 | 0.55 | 0.2 | 0.16 | -0.05 | -0.14 | 0 | 0.04 | 0.35 | 1.21 |
| 0.28 | 0.56 | -0.45 | -0.42 | 0.45 | 0.42 | -0.2 | 0 | -0.34 | -0.39 | -0.28 | 0.03 | 2.21 |
| 0.56 | 0.66 | -0.27 | -0.36 | 0.27 | 0.36 | -0 | -0.2 | 0.06 | -0.13 | -0.1 | -0.09 | 2.21 |
| 0.17 | 0.14 | 0.14 | -0.08 | 0.14 | 0.08 | 0.27 | -0.13 | -0.04 | -0.23 | 0.03 | 0.06 | 2.86 |
| 0.3 | 0.36 | -0.78 | -0.88 | 0.78 | 0.88 | -0.1 | -0.14 | -0.61 | -0.17 | -0.05 | -0.1 | 1.07 |
| 0.75 | 0.68 | -0.11 | 0.07 | 0.11 | 0.07 | 0.22 | 0.03 | 0.1 | -0.04 | 0.07 | 0.03 | 2.5 |
| 0.34 | 0.68 | -0.16 | 0.25 | 0.16 | 0.25 | -0.5 | 0.11 | -0.81 | 1.19 | -0.33 | -0.09 | 2.57 |
| 0.69 | 0.27 | 0.28 | -0.17 | 0.28 | 0.17 | 0.23 | 0.2 | -0.12 | 0.07 | 0.42 | 0.11 | 5.71 |
